# Editorial: Nature and nurture in early onset lung cancer

**DOI:** 10.3389/fonc.2025.1750797

**Published:** 2025-12-04

**Authors:** Ilit Turgeman, Brian S. Henick, Laura Mezquita

**Affiliations:** 1 Medical Oncology, Dana-Farber Cancer Institute, Harvard Medical School, Boston, MA, United States; 2 Department of Medicine, Division of Hematology and Oncology, Columbia University Irving Medical Center, New York, NY, United States; 3 Herbert Irving Comprehensive Cancer Center, Columbia University Irving Medical Center, New York, NY, United States; 4 Department of Medical Oncology, Hospital Clinic of Barcelona, Laboratory of Translational Genomics and Targeted therapies in Solid Tumors, IDIBAPS, Department of Medicine, University of Barcelona, Barcelona, Spain

**Keywords:** young adult oncology, global oncology, precision medicine, immunotherapy, exposome, genetics, environment, microbiome

Lung cancer remains the leading cause of cancer-related mortality worldwide. Although historically linked to older age and tobacco exposure, an increasing number of cases are now being diagnosed in younger individuals that present with distinct clinical, molecular, and psychosocial characteristics. This epidemiologic shift is reshaping our understanding of lung cancer etiology and highlights the need to reconsider the biological drivers and the models of care for this population.

This Research Topic, Nature and Nurture in Early-Onset Lung Cancer (EOLC), brings together contributions that elucidate global patterns of EOLC, explore its clinical and molecular heterogeneity, and discuss how these insights are shaping precision prevention, diagnostics, and therapeutic strategies. Collectively, these articles emphasize the complex interplay between inherited susceptibility (“nature”) and environmental exposures (“nurture”) in the emergence of EOLC.

## Global and regional perspectives on EOLC

The three reviews in this Research Topic (Florez et al., De Guzman, Tansir et al.) converge on a consistent clinical phenotype: patients are more often women, frequently never- or light-smokers, typically have adenocarcinoma, and are commonly diagnosed at advanced stages. These individuals also exhibit a higher prevalence of actionable oncogenic drivers (e.g., *EGFR, ALK* alterations), reinforcing the need for universal molecular profiling, even in low-resource settings.

Florez et al. expand the discussion beyond tumor biology, framing EOLC as both a survivorship and health-equity issue. Younger patients must navigate treatment while facing fertility and sexual-health concerns, interruptions in education and employment, caregiving responsibilities and financial toxicity. The authors call for prospective registries that integrate clinical, molecular, environmental and survivorship data – echoing calls across early-onset cancer literature for biologically informed screening and incorporation of exposomic and multi-omics data through international collaborations (1**).** Moreover, patient voice, education and lived experiences are essential to designing care models that meet their unique needs (2).

De Guzman situates Asia as the geographical epicenter of EOLC, accounting for roughly three quarters of cases worldwide. Beyond the high frequency of targetable alterations, patients are exposed to predominantly non-tobacco carcinogens – ambient and household air pollution, biomass fuel, radon and secondhand smoke, as well as predisposant conditions, such as prior tuberculosis. This review notes correlations between these exposures and molecular alterations such as MUC16 overexpression, and reduced T-cell infiltration, which might attenuate immunotherapy responses. These observations align with data linking fine particulate air pollution to EGFR-driven adenocarcinoma (3), and with genomic studies showing distinct signatures and telomere shortening in never-smoker lung cancer arising in high-pollution regions (4).

Tansir et al. bring an under-represented Indian perspective, noting very low smoking rates but substantial environmental exposure and frequent clinical overlap with tuberculosis, contributing to delayed diagnosis and high rates of metastatic presentation. Limited access to molecular testing and targeted therapy remains a major unmet need (5). Importantly, germline analyses in young Indian patients revealed higher rates of pathogenic variants in DNA-repair pathways (e.g., Fanconi genes, BAP1, etc), consistent with emerging evidence that TP53, BRCA2, other DNA-repair genes and occasionally EGFR T790M germline variants are enriched in young, especially never-smoking patients, supporting routine paired somatic-germline testing in this group (6).

## Expanding the exposome: endocrine, metabolic, and microbial layers

The Mendelian randomization study by Fend et al. introduces a rarely explored dimension: endocrine and metabolic regulation. Hormonal biomarkers, including sex steroids, thyroid and parathyroid hormones, insulin, and prolactin, showed histology-specific associations with lung cancer, with insulin emerging as the only shared factor across adenocarcinoma, squamous, and small-cell subtypes. Conceptually, this positions endocrine and metabolic tone alongside air pollution and biomass as sustained, quantifiable exposures that interact with inherited susceptibility and somatic evolution. Such interactions may help explain the concentration of EOLC in women and in specific ancestries and geographic regions. This is consistent with evidence that have heightened vulnerability driven, at least in part, by hormonal and immunologic factors (7, 8), and it raises further questions about how intrinsic physiological states might interact with exogenous hormonal influences, including diet and hormone-based therapies.

Yang et al. extend the exposome framework to the tumor-associated microbiome. Analysis of tumor and bronchoalveolar lavage fluid across adenocarcinoma progression stages revealed microbial shifts linked to recurrent genomic alterations (e.g., PTPRZ1). This data suggests a model in which the lung microbiota act not as a passive passenger but as an active contributor to early carcinogenesis. This complements evidence across cancer types that microbiome composition modulates tumor initiation, immune tone, and response to PD-1/PD-L1 blockade, and that antibiotics can worsen outcomes.

## Conclusion

This Research Topic places EOLC at the intersection of geography-specific exposures, inherited susceptibility, hormonal and metabolic states, and microbial ecology. It also underscores the impact of health-system disparities on diagnosis, treatment access and survivorship. Young adults with lung cancer face unique medical and psychosocial challenges that demand proactive, age-adapted care models. Addressing these needs will demand not only clinical innovation, but also integration of patient-centered outcomes, equitable access strategies, and sustained attention to fertility, employment, and financial toxicity.

Taken together, these insights support the development of a more integrated framework that combines genomic and epidemiologic research, environmental health initiatives, and equitable models of diagnosis, treatment, and survivorship. Progress in this field will depend on recognizing the uniqueness of this patient population through continued collaboration across regions and disciplines, ensuring that emerging evidence is translated into practice in a coordinated and scalable way.
